# High Producer Haplotype (CAG) of -863C/A, -308G/A and -238G/A Polymorphisms in the Promoter Region of *TNF-α* Gene Associate with Enhanced Apoptosis of Lymphocytes in HIV-1 Subtype C Infected Individuals from North India

**DOI:** 10.1371/journal.pone.0098020

**Published:** 2014-05-16

**Authors:** Sukhvinder Singh, Aman Sharma, Sunil K. Arora

**Affiliations:** 1 Department of Immunopathology, Postgraduate Institute of Medical Education & Research, Chandigarh, India; 2 Department of Internal Medicine, Postgraduate Institute of Medical Education & Research, Chandigarh, India; University of Texas Health Science Center San Antonio Texas, United States of America

## Abstract

**Introduction:**

The natural history of HIV-1 infection and its progression towards AIDS vary considerably among individuals. Host genetic factors may be one of the possible reasons for variable HIV-1 disease progression. Single nucleotide polymorphisms (SNPs) in the promoter region of *TNF-α* gene can influence its production. The aim of the present study was to determine the association of functional *TNF-α* SNPs and its associated parameters related to apoptosis that may influence the rate of HIV-1 disease progression.

**Methods:**

Therapy naive, 100 HIV slow progressors (SPs), 100 HIV fast progressors (FPs), 50 HIV exposed but seronegative individuals (ESNs) and 260 healthy controls from same ethnic origin were recruited. Genotyping of *TNF-α* variants (−863C/A, -308G/A and -238G/A) was done using PCR-RFLP. CD4 counts were determined by flow cytometry. Plasma viral load was estimated by COBAS AMPLICOR HIV-1 monitor test. Plasma TNF-α concentration was estimated by Human CBA Th1/Th2 cytokine kit. The lymphocyte mitochondrial membrane potential was measured by JC-1 dye by flow cytometry.

**Results:**

Genotype and allele frequency of *TNF-α* -238G/A and -863C/A was not significantly different in HIV-1-infected patients when compared to controls, while that of *TNF-α -*308G/A variant (high TNF-α producer) was significantly higher in FPs compared to SPs (*p*<0.01, OR = 3.43). Haplotype analyses also showed that carriers of high TNF-α producing haplotype CAG was significantly more common among FPs compared to SPs (*p*<0.01, OR = 3). The circulating TNF-α levels in blood also correlated well with genotypes. The lymphocyte mitochondrial membrane potential of FPs having CAG haplotype was significantly low as compared to wild type (CGG) haplotype (417±22 vs 571±28, *p*<0.01).

**Conclusion:**

High producer haplotype, CAG of *TNF-α* gene associates with enhanced apoptosis of lymphocytes in HIV-1 infected individuals, hence faster progression to AIDS. However, further functional studies are needed to confirm this association and this knowledge may help clinicians to better understand the disease outcome.

## Introduction

The natural history of HIV-1 infection and its progression towards AIDS vary considerably among individuals. Polymorphism of the ‘immune response genes’ including chemokine receptor/ligand system may be one of the possible reasons for this variable rate of disease progression [1]. Single nucleotide polymorphisms (SNPs) in the promoter region of cytokine genes may affect the quality of the immune response against a pathogen [2]. The impact of such SNPs on the course of HIV-1 infection has not been seriously investigated in Indian population. The tumor necrosis factor-alpha (TNF-α) is a pleiotropic cytokine that acts as an immune and inflammatory mediator, its gene located in the class III region of the major histocompatibility complex (MHC) on chromosome 6. Although its production is tightly regulated *in vivo*, the genotypic differences may influence transcriptional regulation [3]. The excessive TNF-α production is known to be associated with various autoimmune, inflammatory and infectious pathologies [4] [5]. Investigations suggest that TNF-α is involved in the pathogenesis of HIV-1 infection, since it is overproduced by infected individuals [6] [7]. High concentration of TNF-α may influence HIV replication via clonal expansion of infected T lymphocytes. In addition, TNF-α induces rapid apoptosis of bystander T helper cells, thereby accelerating the rate of T-cell depletion in HIV disease [8]. Several functional SNPs have been identified within the *TNF-α* gene cluster, the most widely studied of which are: G>A transition at position -238, G>A transition at position -308, and C>A transversion at position -863 [9], [10], [11]. Few case-control studies have assessed the influence of *TNF-α* gene-polymorphism on HIV-1 disease progression in infected patients [12] [11]. While some of these studies have reported an association with disease progression [13] [14], others have failed to find such an association [15]. There is a lack of comparative data on the influence of *TNF-α* SNPs on various clinical cohorts of HIV-1 infections like fast progressors, elite controllers, long-term non-progressors and exposed but seronegative individuals.

The aim of the present study was to determine the associations, if any, of potentially functional *TNF-α* gene SNPs, both individually and at the haplotype level, with the rate of HIV disease progression or risk of HIV infection in individuals from North India. In addition, we have attempted to explore whether any of these polymorphisms are associated with parameters related to apoptosis that may influence the rate of decline of CD4 cells.

## Materials and Methods

### 1.1 Ethical Statement

The study was approved by the Institutional Ethics Committee (IEC) of PGIMER, Chandigarh, India and an informed consent was obtained from all the patients and healthy controls before obtaining the blood specimen.

### 1.2 Study Design

Patients in different study groups were recruited from ICTC (Intergraded Counseling and Testing Center) in the Department of Immunopathology, PGIMER, Chandigarh, India. The study was carried out on HIV seropositive cohorts from North India that included: Group-I (Slow Progressors; SP, n = 100): therapy naïve individuals who maintained CD4 count of >400 cells/µL for past 3 years or more (slope of decline<-32 cells/µL/Year); Group II (Fast Progressors; FP, n = 100): individuals with fast depletion of CD4 count to <200 cells/µL within one year (slope of decline>-200 cells/µL/Year). Group III (Exposed sero-negative; ESN, n = 50): individuals who were exposed to HIV but remained seronegative. Inclusion criteria in this group was: the HIV seronegative partners of discordant couples, having unprotected sexual intercourse for past one year with a minimum of 4 to 5 exposures every month. The recruitment was based on a detailed history of each couple by the counselor to ascertain the unprotected exposure for a specified period of time and the HIV negative status based on serology as well as HIV-1 DNA PCR. Group IV (Healthy controls; HCs, n = 260): HIV seronegative healthy volunteers from the same geographical region. HIV patients on anti-retroviral therapy (ART) and HIV patients with TB and other chronic co-infections (HCV, HBV) were excluded from the study.

### 1.3 DNA Extraction

Fresh peripheral blood samples (5 mL) were obtained from antecubital vein in evacuated EDTA vials (Vacutainer, BD Biosciences, USA). DNA extraction was carried out using DNA extraction kit (Real Genomics, Banqiao City, Taiwan) from the whole blood. The quality and quantity of DNA were checked spectrophotometerically (Specord 200 spectrophotometer, Analytik Jena, Germany).

### 1.4 Confirmation of HIV Negative Status in ESNs

Absence of pro-viral DNA of HIV-1 was confirmed in each ESN individual using an in-house developed nested-PCR, as described previously [16].

### 1.5 Confirmation of Absence of *CCR5Δ32* Mutation

The most widely known genetic association with 32 bp deletion in the *CCR5* gene (*CCR5Δ32*), which confers nearly complete protection from HIV-1 infection in homozygous individuals was ruled out in ESNs by PCR as described previously [17].

### 1.6 Polymorphism at -238 (rs 361525) and -308 (rs 1800629) Positions in *TNF-α* Gene

This polymorphism consists of a G to A substitution at positions -238 and -308 in the proximal promoter of the *TNF-α* gene. For genotyping nested PCR was carried out resulting in amplification of 266 bp product (spanning -372 bp to -106 bp). The reaction mixture (20 µL) comprised of 50–100 ng of template DNA, 10 pM of each primer (Sigma Genosys, USA), 200 µM dNTP mix (Fermentas, Vilnius, Lithuania) and 1 unit of Taq DNA polymerase (NEB, USA) along with 2 µL of 10X buffer (consisting of 0.1 M Tris-HCl, pH 8.8, 1.5 mM MgCl_2_, 0.5 M KCl and 1% Triton X-100). The primers [18] TNF-P1 F: 5′ –GAA GGA AAC AGA CCA CAG AC-3′ and TNF-P1 R: 5′ –ATC TGG AGG AAG CGG TAG TG-3′ amplified a 266 bp fragment. PCR conditions included initial denaturation at 94°C for 5 min followed by 35 cycles of denaturation at 94°C for 35 Sec, annealing at 57°C for 30 Sec, extension at 72°C for 30 Sec, with final extension for 5 min at 72°C. PCR was carried on a Master cycler gradient machine (Eppendorf, Hamburg, Germany). The PCR products were monitored by gel electrophoresis in a 2% agarose gel (containing 0.5 µg/mL ethidium bromide). DNA bands were visualized under UV light and digitally photographed using a gel documentation system (Syngene, Cambridge, England). Amplified products of first (external) PCR were used as template in the second (internal or nested) PCR to amplify 118 bp products. Nested PCR was carried out by using primers: TNF-Nc F: 5′-AGG CAA TAG GTT TTC AGG TCC ATG-3′ and TNF-Bg R: 5′–CAC ACT CCC CAT CCT CCC AGA TC-3′. The PCR conditions were same as mentioned above. Four micrograms of nested PCR product (118 bp) was digested at 37°C for 8 hours in a 10 µL reaction volume containing 2.5 units of restriction enzyme *Nco*I (NEB, USA) to detect -308G/A polymorphism [18] and 2.5 units of restriction enzyme *Bgl*II (NEB, USA) to detect -238G/A polymorphism, and then analyzed on a 15% non-denaturing polyacrylamide gel (PAGE). Restriction fragment length polymorphism (RFLP) analysis for -308G/A SNP resulted in two bands (94 and 24 bp) for GG genotype, three bands (118, 94 and 24 bp) for GA heterozygous and single uncut band (118 bp) corresponding to AA genotype. Similarly RFLP analysis for -238G/A SNP resulted in two bands (95 and 23 bp) for AA genotype, three bands (118, 95 and 23 bp) for GA heterozygous and single uncut band (118 bp) corresponding to GG genotype.

### 1.7 Polymorphism at -863 Position in *TNF-α* Gene (rs 1800630)

This polymorphism consists of a C to A substitution at position -863 in the proximal promoter of the *TNF-α* gene. The subjects were genotyped for this polymorphism by PCR–RFLP as described previously [19]. The region surrounding the polymorphism was amplified using forward primer F:5′-GGC TCT GAG GAA TGG GTT AC-3′ and reverse primer R:5′-CTA CAT GGC CCT GTC TTC GTT ACG-3′. Amplification was performed in a reaction volume of 20 µL containing 1 mM MgCl_2_, 0.2 mM of each nucleotide (Fermentas, Vilnius, Lithuania), and 1 U of Taq DNA polymerase (NEB, USA). PCR conditions included initial denaturation at 94°C for 5 min followed by 35 cycles of denaturation at 94°C for 30 Sec, annealing at 62°C for 1 min and extension at 72°C for 30 Sec, the final extension being at 72°C for 5 min. Four micrograms of amplified product (126 bp) was then digested with *Bsa*AI restriction enzyme (NEB, USA) at 37°C for 8 hours, electrophoresed on 15% PAGE at 60 V and stained with ethidium bromide. The 126 bp band corresponded to the C wild-type C allele and a set of 103 bp and 23 bp bands corresponded to the variant A allele.

### 1.8 Plasma TNF-α Level Estimation

Plasma TNF-α concentration was estimated by Human CBA Th1/Th2 cytokine kit (Cytometric Bead Array, BD Biosciences, San Jose, CA, USA) according to the manufacturer’s instructions. The lower limit of TNF-α detection using this kit was 2.8 pg/mL. Standard curves generated and plasma TNF-α concentrations were calculated using the BD CBA Software (BD Bioscience, San Jose, CA, USA).

### 1.9 Assessment of Blood CD4+ T-cell Count and Plasma Viral Load

Absolute CD4 T-lymphocyte counts were estimated in the whole blood by flow cytometry using BD Tritest CD3 FITC/CD4 PE/CD45 PerCP with BD Trucount tubes (BD Biosciences, San Jose, CA, USA) and plasma viral load (pVL) was estimated using COBAS Amplicor HIV-1 monitor test, version 1.5 (Roche, NJ, USA) as per the manufacturer’s guidelines.

### 1.10 Mitochondrial Membrane Potential (Δψ_m_) Estimation

Mitochondrial membrane potential (Δψm) was measured flow cytometrically using 5, 5′, 6, 6′-tetraethylbenzimidazolocarbocyanine iodide (JC-1) dye (Sigma-Aldrich, USA) [20]. Briefly, 2 mL of EDTA anti-coaggulated whole blood was treated with RBC lysis buffer (Himedia, Mumbai, India) and leukocytes were washed with phosphate buffer saline (PBS, Himedia, Mumbai, India). Cell count was adjusted to 1×10^6^ cells/mL before staining with JC-1 dye (final concentration 2.5 µM) by incubation at 37°C (5% CO_2_ humidified atmosphere) for 15 minutes in dark. Stained cells were washed with PBS and acquired immediately on flow cytometer (FACSCanto II). The data was analysed using software FACSDiva Version 6.1.3 (BD Biosciences, USA). As a positive control for maximum depolarization of mitochondrial membranes, the mitochondrial uncoupling agent protonophore carbonyl cyanide p-(trifluoromethoxy) phenylhydrazone (FCCP) (Sigma-Aldrich, USA) was used at final concentration of 2 µM. A total of 50,000 events were acquired for each assayed sample. Lymphocytes were gated on forward and side scatter to exclude debris and non-lymphoid cells. JC-1 fluorescence was analysed on FL1 and FL2 channels for the detection of dye monomers (Green) and J-aggregates (Red), respectively. The ratio of red/green fluorescence reflected mitochondrial trans-membrane potential (Δψm). The treatment with the protonophore FCCP resulted in decreased JC-1 fluorescence ratio and served as a positive control for disruption of mitochondrial membrane causing change in membrane potential. The Δψm is expressed as percent median fluorescence intensity (FL2:FL1/FL2 FCCP: FL1 FCCP)×100.

### 1.11 Statistical Analysis

Statistical analysis was performed using GraphPad Prism version 5.0 (GraphPad Software, Inc.). Power analysis was performed using Quanto software (version 1.0; http://hydra.usc.edu/gxe). Hardy-Weinberg equilibrium (HWE) was examined for each SNP by online HWE calculator (http://www.oege.org/software/hwe-mr-calc.shtml). The frequencies of genotypes and alleles were compared by Fisher’s exact test. Fisher’s exact test was also used to analyze association between HIV disease progression and *TNF-α* variants. Odds ratio (OR) and its 99% confidence interval (C.I.) were calculated to assess the risk conferred by a particular allele and genotype. Haplotype analysis was performed using PHASE (http://www.stat.washington.edu/stephens/software.html) v2.1. Slope of CD4 T cell decline was calculated by linear regression model. Discrete and continuous variables were compared between cases and control subjects using unpaired Student’s t-test, Two way ANOVA and Mann–Whitney test as appropriate. The Bonferroni correction for multiple testing was applied when necessary. A two sided *p*-value of <0.05 was considered statistically significant.

## Results

A total of 260 healthy volunteers (HIV-1 seronegative, HCs), 200 HIV-1 seropositive patients and 50 ESNs were genotyped for -863C/A, -308G/A, and 238G/A polymorphisms in the promoter region of *TNF-α* gene. Genotype distribution and allelic frequency were compared between HCs, SPs, FPs and ESNs. The distribution of the genotype frequencies at these loci were found to be in Hardy–Weinberg equilibrium.

### 3.1 Characteristics of Study Population

All individuals (510) included in this study were ethnically from the North Indian states. The demographic and clinical profile of individuals in different study groups is summarized in Table 1. No significant difference was found in the age and sex distribution between these study groups. All ESNs were negative for HIV-1 infection by serology as well as by DNA PCR and none of them was positive for the presence of *CCR5Δ32* mutation (data not shown). For the HIV-1 seropositive individuals included in this study, we had the baseline values of viral load and CD4 count both at the time of registration in the ART clinic as well as at the time of recruitment in this study. At the time of registration the FPs had initially higher median CD4 count than SPs (617 vs 583 cells/µL, Range given in Table 1), but within one year there was a steep decline in CD4 count in FP group (slope of decline for CD4 count >-200 cells/µL/Year, mean -464 cells/µL/Year, Range -201 to -506 cells/µL/Year), whereas in SP group the rate of decline was only marginal (slope of decline <-32 cells/µL/Year, mean -18 cells/µL/Year, range 20 to -32 cells/µL/Year) even after more than three years of HIV detection. Significant difference was found in the slope of CD4 count decline in FPs as compared to SPs (Unpaired T-test, *p*<0.0001). The HIV plasma mean log viral load was also significantly (Unpaired T-test, *p*<0.0001) higher in FPs (5.4 Log_10_ RNA copies/mL) as compared to SPs (3.4 Log_10_ RNA copies/mL, range given in Table 1).

**Table 1:** Demographic and clinical characteristics of the population analyzed.

Characteristic	HCs (n = 260)	SPs (n = 100)	FPs (n = 100)	ESNs (n = 50)
Sex (M/F ratio)	1.05	1.2^a^	1.1^b^	0.87^c^
Age (years) (median, range)	27 (19–48)	34.5 (18–52)	35 (21–51)	33 (24–42)
Duration of HIV-1 infection (months) (mean)	–	60 (38–96)	12 (8–14)	–
CD4 T-cell count (cells/µl) (median and range) at the time of HIV Diagnosis	–	583 (419–1116)	617 (417–733)	–
CD4 T-cell count (cells/µl) (median and range) at the time of recruitment	862.5 (463–1471)	495 (411–884)||	202 (142–227)||	600 (465–884)
Plasma HIV-1 RNA (copies/ml) (mean Log Value and range)	–	3.4 (2.7–4.1)#	5.4 (4.87–5.85)#	–
Exposure to HIV-1 History				
Sexual	–	82 (%)	88 (%)	–
Blood Transfusion	–	4 (%)	2 (%)	–
Intravenous Drug User	–	2 (%)	3 (%)	–
Other	–	12 (%)	7 (%)	–

a, SPs vs HCs, *p*>0.05; b, FPs vs HCs, *p*>0.05; c, ESNs vs HCs, *p*>0.05.

# Viral Load, FPs vs SPs, *p*<0.0001.

|| CD4 Count, FPs vs SPs, *p*<0.0001.

### 3.2 *TNF-α* Gene -238G/A Polymorphism

**Figure 1:**
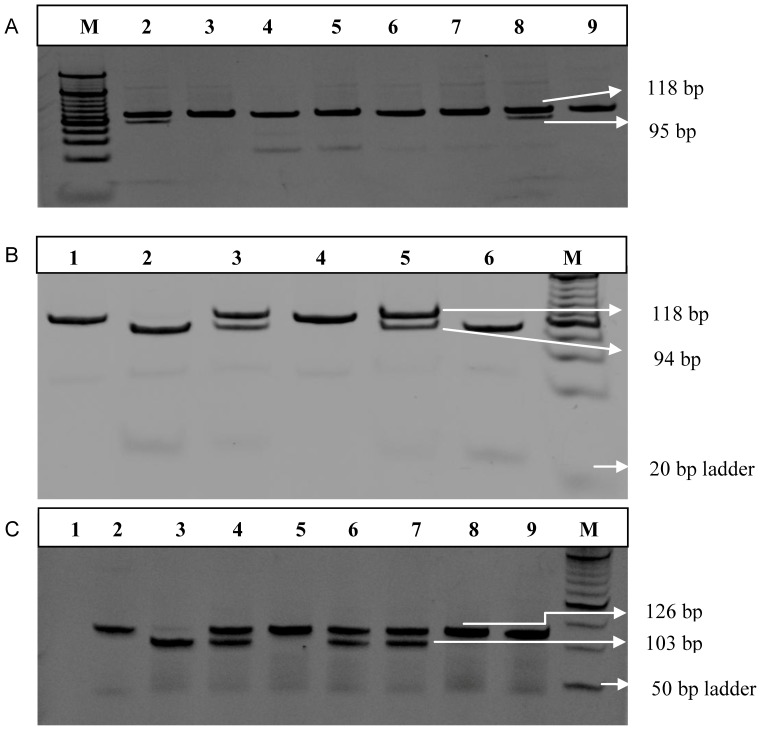
Restriction fragment length polymorphism (RFLP) analysis of PCR products. (A) Representative ethidium bromide stained (15%) non-denaturing polyacrylamide gel (PAGE) showing amplified gene product of *TNF-α* (−238G/A) 118 bp uncut and RFLP pattern observed after digestion with *Bgl*II. (Lane M -20 bp ladder, Lane 2 and 8-GA genotype, Lane 3 to 7-GG Genotype. (B) Representative non-denaturing PAGE (15%) showing amplified gene segment of *TNF-α* (−308G/A) 118 bp uncut and RFLP pattern observed after digestion with *Nco*I. Lane 1-uncut 118 bp product, Lane 2 and 6-GG Genotype, Lane 3 and 5-GA genotype, Lane 4-AA Genotype, Lane M-20 bp Ladder. (C) Representative non-denaturing PAGE (15%) showing amplified gene segment of *TNF-α* (−863C/A) 126 bp uncut and RFLP pattern observed after digestion with *Bsa*AI. Lane 1-Negative Control, Lane 2-uncut 126 bp, Lane 3-AA Genotype, Lane 4-CA Genotype, Lane 5, 8 and 9-CC Genotype, Lane 6 and 7-CA Genotype, Lane M-50 bp ladder.

On genotyping of -238G/A polymorphism (Figure 1A) in the promoter region of *TNF-α* gene we found genotype distribution of GG (91%) and GA (9%) in the healthy controls with allele frequency of G allele at 95.38% and A allele at 4.62%, which was considered as wild type distribution (Table 2). As compared to this no significant difference was found in genotype and allele frequency of this polymorphism in HIV-1 seropositive individuals. Similarly there was variation in distribution but difference was not significant within HIV seropositive groups viz. SPs vs FPs or ESNs group (Table 3).

**Table 2:** *TNF-α* genotype and allele frequencies in HCs, HIV-1 infected (FPs+SPs) and ESNs.

Genotype and allele frequencies	HCs	HIV-1 n = 200 (%)		HIV-1 vs HCs		ESNs	HIV-1 vs ESNs	
*TNF-α* (−238G/A)	n = 260 (%)	SPs (n = 100)	FPs (n = 100)	*p* value	OR (95% CI)	n = 50 (%)	*p* value	OR (95% CI)
GG	236 (91)	93 (93)	88 (88)	Ref.		46 (92)	Ref.	
GA	24 (9)	7 (7)	12 (12)	1	1.03 (0.54–1.94)	4 (8)	1	0.85 (0.28–2.58)
G	496 (95.38)	193 (96.5)	188 (94)	Ref.		96 (96)	Ref.	
A	24 (4.62)	7 (3.5)	12 (6)	1	1.03 (0.55–1.96)	4 (4)	1	0.86 (0.29–2.53)
***TNF-α*** ** (**−**308G/A)**								
GG	234 (90)	94 (94)	82 (82)	Ref.		48 (96)	Ref.	
GA	23 (9)	6 (6)	17 (17)	0.43	1.33 (0.72–2.44)	2 (4)	0.39	0.42 (0.09–1.85)
AA	3 (1)	0	1 (1)	0.54	1.22 (0.68–2.21)	0	NC	
G	491(94.42)	194 (97)	181 (90.5)	Ref.		98 (98)	Ref.	
A	29 (5.58)	6 (3)	19 (9.5)	0.67	1.12 (0.65–1.96)	2 (2)	0.2	0.34 (0.08–1.42)
***TNF-α*** ** (**−**863C/A)**								
CC	130 (50)	48 (48)	51 (51)	Ref.		23 (46)	Ref.	
CA	114 (43.85)	45 (45)	45 (45)	0.92	1.03 (0.70–1.51)	24 (48)	0.63	1.19 (0.63–2.22)
AA	16 (6.15)	7 (7)	4 (4)	0.84	0.84 (0.40–2.03)	3 (6)	1	1.06 (0.28–3.93)
C	374 (71.92)	141 (70.5)	147 (73.5)	Ref.		70 (70)	Ref.	
A	146 (28.08)	59 (29.5)	53 (26.5)	1	0.99 (0.74–1.33)	30 (30)	0.71	0.90 (0.56–1.46)

Data represented as n = number of subjects, Number in parentheses gives the data in percentage.

Data represented as OR (95% CI), odds ratio (confidence interval), Pearson χ2 or Fisher exact test was performed to determine group differences.

**Table 3:** Comparisons of *TNF-α* genotype and alleles in SPs, FPs and ESNs with HCs associations with HIV disease progression.

*TNF-α* (238G/A)	SPs vs HCs		FPs vs HCs		ESN vs HC		FPs vs SPs		FP vs ESNs	
	*p* value	OR (99% CI)	*p* value	OR (99% CI)	*p* value	OR (99% CI)	*p* value	OR (99% CI)	*p* value	OR (99% CI)
**GG**	Ref.		Ref.		Ref.		Ref.		Ref.	
**GA**	0.67	0.74 (0.23–2.34)	0.43	1.34 (0.51–3.52)	1	0.85 (0.20–3.65)	0.33	1.81 (0.50–6.54)	0.58	1.56 (0.32–7.46)
**G**	Ref.		Ref.		Ref.		Ref.		Ref.	
**A**	0.68	0.74 (0.24–2.36)	0.44	1.31 (0.51–3.36)	1	0.86 (0.20–3.56)	0.34	1.76 (0.50–6.16)	0.59	1.53 (0.33–7.02)
**(–308G/A)**										
**GG**	Ref.		Ref.		Ref.		Ref.		Ref.	
**GA+AA**	0.30	0.57 (0.17–1.92)	0.04	1.97 (0.83–4.65)	0.27	0.37 (0.05–2.59)	**0.01**	3.43 (0.96–12.3)	0.02	5.26 (0.72–38.04)
**G**	Ref.		Ref.		Ref.		Ref.		Ref.	
**A**	0.17	0.52 (0.16–1.69)	0.06	1.77 (0.80–3.92)	0.20	0.34 (0.05–2.32)	**0.01**	3.39 (0.98–11.6)	**0.01**	5.10 (0.73–35.89)
**(–863C/A)**										
**CC**	Ref.		Ref.		Ref.		Ref.		Ref.	
**CA**	0.80	1.06 (0.57–2.90)	1	1.00 (0.54–1.87)	0.63	1.19 (0.52–2.70)	0.88	0.94 (0.44–1.99)	0.72	0.84 (0.33–2.11)
**AA**	0.80	1.18 (0.34–4.12)	0.59	0.63 (0.14–2.86)	1	1.00 (0.18–5.93)	0.52	0.53 (0.09–2.93)	0.67	0.60 (0.07–4.77)
**C**	Ref.		Ref.		Ref.		Ref.		Ref.	
**A**	0.71	1.07 (0.66–1.71)	0.71	0.92 (0.56–1.49)	0.71	1.09 (0.59–2.03)	0.57	0.86 (0.48–1.53)	0.58	0.84 (0.41–1.69)

Data represented as OR (99% CI), odds ratio (confidence interval), Pearson χ2 or Fisher exact test was performed to determine group differences.

Statistical significance was considered at *p* value <0.01 due to Bonferroni’s correction for multiple testing.

### 3.3 *TNF-α* Gene -308G/A Polymorphism

The genotype distribution of GG (90%), GA (9%) and AA (1%) was observed in healthy controls with allele frequency for G allele as 92.42% and for A allele 5.58%. No significant difference was found in genotype and allele frequency when compared with overall HIV-1 seropositive group (Table 2). However, the frequency of GA genotype, which is known to be associated with higher production of TNF-α cytokine, was significantly higher in fast progressors (FPs) as compared to slow progressor (SPs) group (*p*<0.01, OR = 3.43). Further comparison between allele frequencies between FPs vs SPs also showed A allele to be significantly over-represented in FPs (*p*<0.01, OR = 3.39) (Table 3). RFLP analysis observed on genotyping *TNF-α* -308G/A is shown in Figure 1B.

### 3.4 *TNF-α* Gene -863 C/A Polymorphism

Genotype analysis of *TNF-α* -863C/A SNP revealed, CC (50%), CA (43.85%) and AA (6.15%) in healthy controls with frequency of C allele as 71.92% and of A allele as 28.08%. No significant difference was observed in the frequency distribution of these alleles among HIV positive groups when compared to HC (Table 3, Figure 1C).

### 3.5 Haplotype Association with Disease Progression

In order to evaluate the combined effects of the three SNPs in the *TNF-α* gene, haplotype analysis was conducted using PHASE software. Of the 8 haplotypes observed in the patients and controls recruited for the study, only three haplotypes: CGG, CAG and AGG were represented at frequencies >5%. These accounted for more than 99% of the total haplotypes. Among these, the frequency of wild type haplotype CGG in healthy controls (65%) was not significantly different from that in SPs (68%). However, the CAG haplotype, which is high producer of TNF-α, was significantly overrepresented in FPs [*p*<0.03, OR = 1.93, 99% CI (0.88–4.21)] as compared to HCs and SPs [*p*<0.01, OR = 3.00, 99% CI (0.98–9.20)] (Table 4). The frequency of low producer haplotype AGG however, was not significantly different among various study groups.

**Table 4:** Observed *TNF-α* haplotypes and disease progression in HIV-1 patients.

Haplotypes *TNF-α*	HCs	FPs	OR (99% CI)	*p* value	SPs	OR (99% CI)	*p* value	OR (99% CI)	*p* value
			FPs vs HCs			FPs vs SPs		SPs vs HCs	
**CGG**	334	117	Ref.		134	Ref.		Ref.	
**CAG**	31	21	1.93 (0.88–4.21)	0.03	8	3.00 (0.98–9.20)	**0.01**	0.64 (0.22–1.84)	0.35
**AGG**	133	54	1.15 (0.70–1.91)	0.49	55	1.12 (0.62–2.03)	0.64	1.03 (0.63–1.68)	0.92

Data represented as OR (99% CI), odds ratio (confidence interval), Pearson χ2 or Fisher exact test was performed to determine group differences. Statistical significance was considered at *p* value <0.016 due to Bonferroni’s correction for multiple testing.

### 3.6 Plasma TNF-α Concentrations

**Figure 2:**
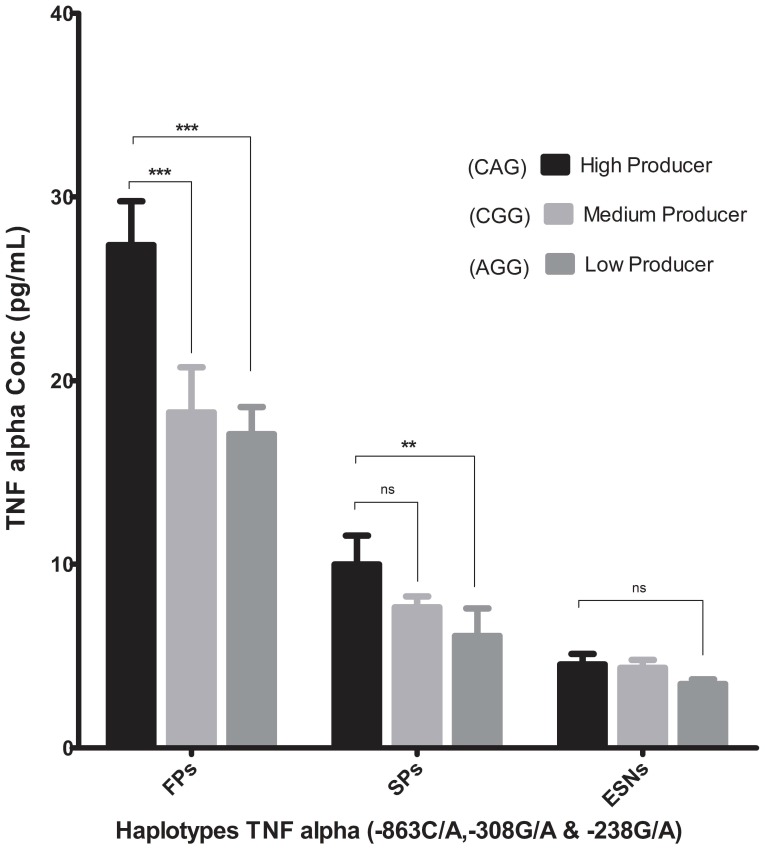
Plasma concentrations of TNF-α in various study groups. Plasma concentrations of TNF-α (Mean±SEM) in FPs, SPs and ESNs. High TNF-α producing haplotype CAG (dark bar), medium TNF-α producer haplotype CGG (light gray bar) and low TNF-α producing haplotype AGG (gray bar).

TNF-α levels were measured in the plasma samples of 18 subjects in each group. The plasma TNF-α levels were significantly (*p*<0.001) higher in FPs (20.9±1.4 pg/mL) as compared to SPs (7.9±1.1 pg/mL) and ESNs (4.1±0.6 pg/mL, *p*<0.0001). We further investigated the effect of haplotypes on TNF-α levels within FP group. Individuals having CAG (high producer) haplotype had significantly (*p*<0.01) higher levels (Mean, 27.36±2.4 pg/mL) of TNF-α as compared to carriers of low producer AGG (17±1.5, pg/mL) & wild type CGG haplotype (18.26±2.4 pg/mL, *p*<0.01) (Figure 2). However, the comparison between haplotypes within SP group showed a significant difference between high producer and low producer haplotypes only (CAG vs AGG: 9.98±1.6 pg/mL vs 6.08±1.5 pg/mL, *p*<0.01). Further, in FPs, positive correlation was also observed between plasma TNF-α concentration and plasma viral load (Pearson correlation, r = 0.77, *p*<0.0002).

### 3.7 *TNF-α* Genotypes and Clinical Parameters

**Figure 3:**
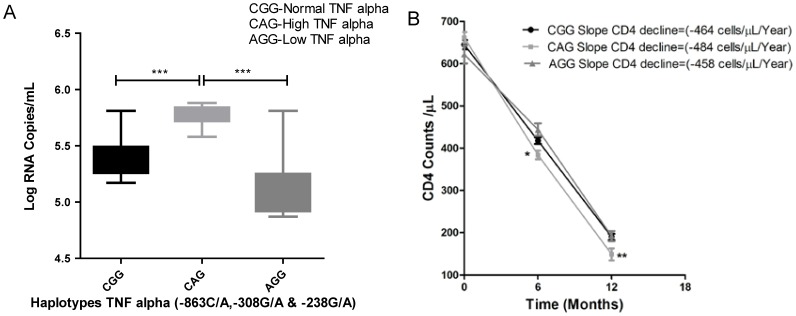
Plasma viral load and CD4 counts in different *TNF-α* haplotypes (CGG, CAG and AGG). (**A**) Plasma Log_10_ RNA copies/mL (Mean±Range) in *TNF-α* haplotypes, CGG (dark bar), CAG (light gray bar) and AGG (gray bar). (**B**) CD4 counts at different time intervals in *TNF-α* haplotypes, CGG (dark bar), CAG (light gray bar) and AGG (gray bar). Values shown as (Median±SD). Significance levels: **p<0.01; *p<0.05.

We tried to investigate the distribution of *TNF-α* haplotypes and baseline viral load and CD4 T-cell counts within FP group. High TNF-α producing haplotype CAG within FP group had significantly higher mean log viral load as compared to carriers of wild type haplotype (CGG) (Mean, 5.8 vs 5.4 Log_10_ RNA copies/mL, Unpaired T-test, *p*<0.001) as well as low producer AGG and faster decline of CD4 counts as observed by steeper decline of CD4 slope (mean slope of decline for CD4 count -484 cells/µL/Year vs -464 cells/µL/Year) (Unpaired T-test, *p*<0.01) (Figure 3 A, B).

### 3.8 Haplotypes and Mitochondrial Membrane Potential

**Figure 4:**
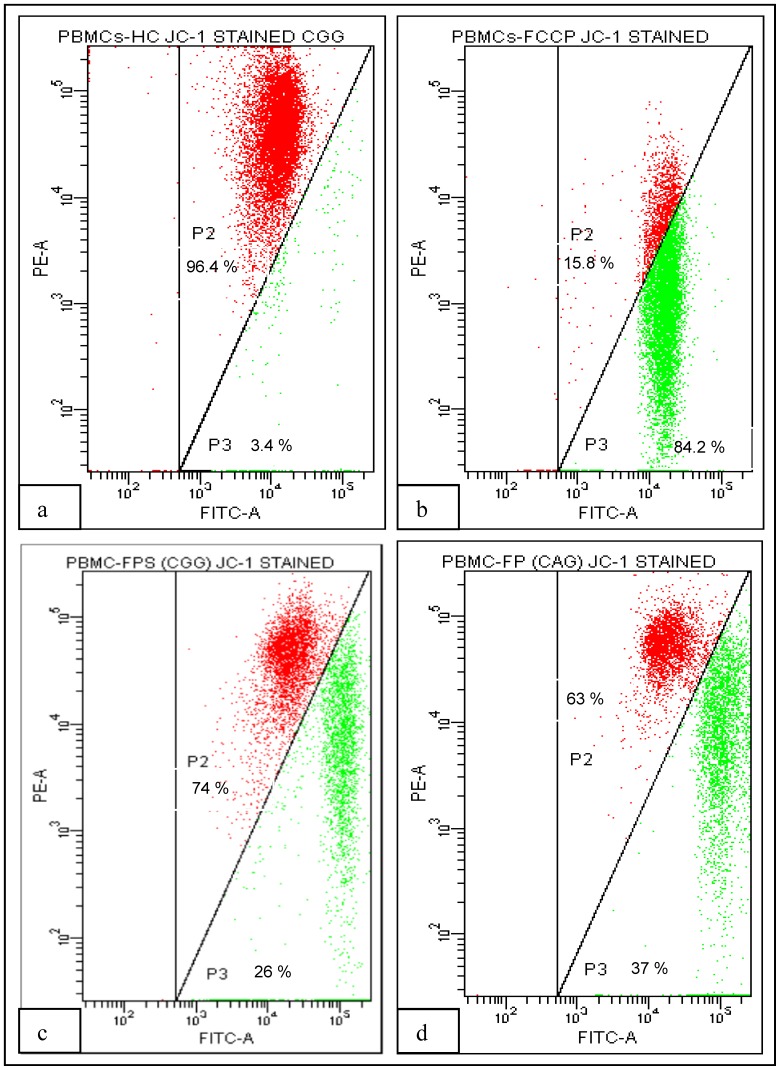
Gating strategy for the analysis of JC-1 stained PBMCs. Lymphocytes were gated according to morphological parameters (not shown in figure), JC-1 aggregates and monomers were analyzed on FL2 (PE) and FL1 (FITC) channel respectively, (a) showing JC-1 stained lymphocytes of HCs (P2 gate, 96.4%), (b) protonophore FCCP treated lymphocytes as a positive control showing 84.2% cells having reduced mitochondrial membrane potential. (c) and (d) JC-1 stained lymphocytes in a representative samples from individuals in ‘fast progressors’ group having CGG and CAG haplotypes showing 26% and 37% lymphocytes dying respectively. Change in mitochondrial membrane potential (Δψm) was expressed as percentage of median fluorescence intensity (FL2: FL1/FL2 FCCP: FL1 FCCP) x100.

**Figure 5:**
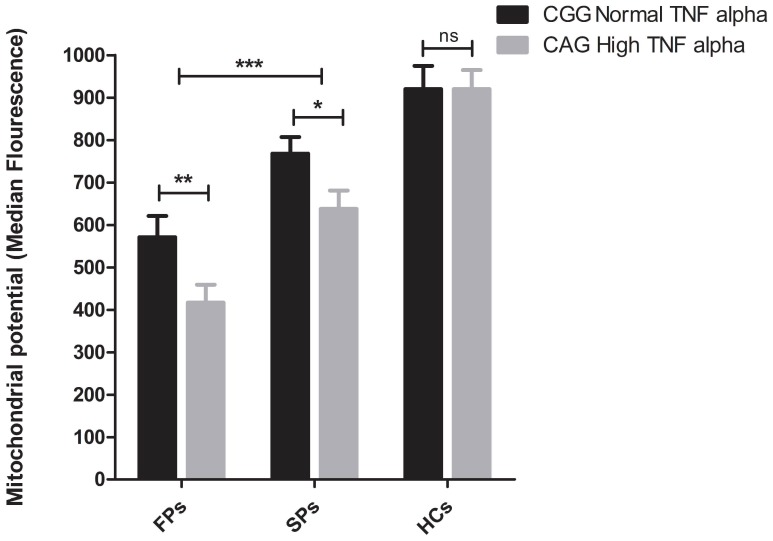
Median fluorescence intensities of total lymphocyte mitochondrial membrane potential (Δψm). Bar diagram showing median fluorescence intensity (MFI) of total lymphocyte mitochondrial membrane potential (Δψm) in FPs and SPs having CAG (n = 5) (dark bar) and CGG (n = 5) (light gray bar) haplotypes (shown as Mean±SEM).

Since, the high producer genotypes and haplotypes combined with high plasma TNF-α concentration were found to be associated with faster decline in CD4 count, we investigated further, whether these genotypic differences could influence the apoptosis of lymphocytes in terms of decrease in mitochondrial membrane potential (Δψm) in HIV infected individuals (Figure 4). In order to address that, subjects (n = 5) were recruited in each group based on the haplotypes [CAG (high TNF-α producer) vs CGG (low TNF-α producer)]. Significantly low mean Δψm was observed in HIV-1 infected patients as compared to healthy controls (669±34 vs 920±45, Unpaired T-test, *p*<0.003). Also, Δψm was significantly lower in FP group as compared to SP group (493±25 vs 703±24, Unpaired T-test *p*<0.002). Further, when we compared Δψm of different haplotypes within each group or between groups (Figure 5), we found that individuals having CAG haplotype had significantly lower mean Δψm as compared to carriers of wild type (CGG) haplotype within FP group (417±22 vs 571±28, Two way ANOVA, *p*<0.01), indicating higher cell death among high TNF-α producing haplotype carriers as compared to wild type. Similarly in case of SP group also, individuals having CAG haplotype had lower Δψm as compared to carriers of wild type (CGG) (638±19 vs 768±29, Two way ANOVA, *p<*0.05).

## Discussion

The present study evaluated the frequency of *TNF-α* promoter polymorphisms and their association with HIV-1 disease progression or risk of HIV infection, plasma levels of TNF-α and mitochondrial membrane potential in ethnically defined North Indian population. The *TNF-α* gene is located within the class III region of the major histocompatibility complex (MHC) between HLA-B and DR, and its expression in the host is controlled in a regulated manner at the transcriptional as well as post transcriptional level. Polymorphism in the *TNF-α* promoter region has been shown to play an important role in the development of autoimmune diseases and several infections like HBV, HCV, leishmaniasis, etc. [5] [21] [22], [23]. The -308A allele in the *TNF-α* promoter is a G/A transition and is the most studied SNP in this gene. Our data indicate that *TNF-α* -308 G/A polymorphism is associated with faster progression of HIV disease both at genotype and allele frequency level. Although, when Bonferroni’s corrections are applied for multiple comparisons, the significance level comes at border line only(corrected *p* values: *p*<0.01, OR = 3.43, *p*<0.01, OR = 3.39). Haplotype analyses also suggest that the combination of some genetic variants within the *TNF-α* gene (e.g. CAG haplotype), conforming to higher production of the cytokine was represented in significantly higher number of FPs as compared to SPs, suggesting that the high producers of TNF-α would show faster disease progression. These results do agree with some previous reports, which have shown an association between *TNF-α* SNPs -238G/A and -308G/A and the risk of HIV-1 infection [13] [14] while not with others which observed no significant association [15]. This discrepancy may be attributed to the different ethnic background of assessed populations in these studies.

Furthermore, we observed a significantly lower mean lymphocyte mitochondrial membrane potential (Δψm, an indicator of cell apoptosis) in FPs as compared to SPs and even within group (FPs) carriers of CAG haplotype as compared to carriers of wild type haplotype (CGG), indicating that the individuals having high TNF-α producing genetic background will have faster disease progression due to predisposition to higher rate of cell death.

In the current study, we have found almost similar *TNF-α* -238G/A and -308G/A allele frequencies as reported earlier from North Indian population in studies related to *Mycobacterium tuberculosis* infection and Type-1 Diabetes [24] [25]. It seems therefore that -238A is a rare allele in Indian population (with frequency varying between 2 to 5%) as compared to European population which has been reported to be more than 8% [19]. It is also important to note that the population frequency of *TNF-α* -308A in the Caucasian population is around 17–22%, which is higher than that observed in the Asian populations (5–6%), which is rare as compared to -308G allele (found in 93%), suggesting large variation in the allele frequencies among different ethnic groups [26]. We did not find any significant difference between the frequencies of any of the *TNF-α* alleles or haplotypes in the ESNs and HIV infected groups, indicating no association of these polymorphisms with risk of HIV infection in North Indian cohorts. In contrast to our results, a recent study from Western India (Mumbai) showed a higher frequency of -238GG genotype in exposed uninfected (EU) individuals as compared to those who acquired infection, indicating a possible association with HIV transmission [27]. Another study on Spaniard cohort showed no association with any of the individual polymorphisms at -238(G/A), -308(G/A) and -863(C/A) loci with the vulnerability to HIV infection, however the haplotype analysis in their study indicated that haplotype GAC was more frequently represented in the EU, suggesting the combination of polymorphisms within *TNF-α* gene may positively modulate the risk of HIV infection in an individual [19]. The discrepancy in findings may be due to the ethinic differences in the studied populations. So, further studies at genotype and haplotype levels are needed for a logical explanation regarding association of *TNF-α* polymorphism with risk of infection.

Similar observations have been made in relation to different infections, like in case of HBV infection, the presence of -308A and -863C was found to be associated with viral clearance [5], whereas SNP -238G has been shown to be associated with protection against chronic hepatitis in the Chinese and German population. In hepatitis C virus infection, the haplotype -863C/−308G was associated with viral persistence in African Americans. Similarly the existence of -308A SNP in pediatric population positively correlated with the severity of dengue viral infection in children [28]. With regard to HIV-1 infection, studies have shown association of *TNF-α* -308A allele with HIV-1-associated dementia and other HIV therapy related complications like Lipodystrophy Syndrome in infected patients [10].

The immuno-regulatory response of the host influences the pathogenesis of HIV-1 infection, triggering monocytes, macrophages, and natural killer cells to produce TNF-α [29]. We have found significantly high TNF-α levels in plasma of FPs as compared to SPs and ESNs and there is a positive correlation between HIV-1 viremia and TNF-α levels in plasma of HIV-1 infected individuals. This suggests that reducing TNF-α levels may also help in reducing the HIV-1 viral load. In excess, TNF-α may cause severe inflammatory damage and toxicity, making control of its production and secretion highly important. Regulating its release serves as a potential means of therapy for HIV-1 and other diseases. TNF-α can also induce other pro-inflammatory cytokines such as IL-6 and IL-8, which aid in the up-regulation of viral replication [30] [31]. TNF-α is secreted during the early phase of acute inflammatory diseases. Its pathogenic role in HIV-1 infection involves activation of nuclear factor kB (NF-kB), stimulating viral replication as well as apoptosis of T lymphocytes, both the factors contributing to faster disease progression. Tissue and plasma samples of hosts express high levels of TNF-α, contributing to fever, anorexia, and other symptoms of HIV/AIDS. These observations suggest that TNF-α must be targeted at an appropriate time during the disease to prevent its progression to the chronic stage. Local effect of the cytokine may be beneficial to the host, so monitoring its development is critical.

The current study is novel in terms of our study population as the three highly unique HIV-1 subtype-C infected cohorts from a previously not seriously investigated ethnic population, were studied for the expression of polymorphic alleles that could serve as clinical biomarkers for the progression of HIV-1 infection. To the best of our knowledge this is the first study from North Indian population on *TNF-α* promoter gene polymorphism in HIV-1 infection having different clinical forms. Also, we have tried to find association at the haplotype level as haplotype-based approaches are more powerful than single SNP approaches in the setting of multiple susceptibility loci. However, additional multi-centric studies with larger number of patients from similar cohorts are warranted to establish the detailed mechanisms underlying the functional effects of these genetic polymorphisms. Also, HLA typing of these HIV-1 infected patients is required as recently in genome wide association studies (GWAS) it has been shown that the *TNF-α* SNPs are in linkage disequilibrium with MHC class I gene. It becomes more important in the light of well documented association of some HLA types with the rate of HIV disease progression [32], [33]. None-the-less the data given in this study provides significant information regarding genetic association with progression of disease, which might be very helpful in designing control strategies in such populations.
